# Entecavir competitively inhibits deoxyguanosine and deoxyadenosine phosphorylation in isolated mitochondria and the perfused rat heart

**DOI:** 10.1016/j.jbc.2022.101876

**Published:** 2022-03-28

**Authors:** Avery S. Ward, Chia-Heng Hsiung, Daniel G. Kesterson, Vasudeva G. Kamath, Edward E. McKee

**Affiliations:** 1 Department of Foundational Sciences, College of Medicine, Central Michigan University, Mount Pleasant, Michigan, USA; 2 School of Science, Westlake Institute for Advanced Study, Westlake University, Hangzhou, Zhejiang Province, China; 3 Department of Health Management and Policy, University of Michigan School of Public Health, University of Michigan, Ann Arbor, Michigan, USA; 4 Department of Basic Medical Sciences, Touro College of Osteopathic Medicine, Middletown, New York, USA

**Keywords:** mitochondria, mitochondrial disease, nucleoside/nucleotide biosynthesis, nucleoside/nucleotide metabolism, Ap5A, P1,P5-di(adenosine-5) pentaphosphate pentasodium salt, AZT, 3′-azido-3′-deoxythymidine, dA, deoxyadenosine, dCK, deoxycytidine kinase, dG, deoxyguanosine, dGK, deoxyguanosine kinase, EHNA, erythro-9-Amino-β-hexyl--α-methyl-9H-purine-9-ethanol hydrochloride, erythro-9-(2-Hydroxy-3-nonyl)-adenine hydrochloride, ETV, entecavir, MDS, mitochondrial DNA depletion syndrome, mtDNA, mitochondrial DNA, NRTI, nucleoside reverse transcriptase inhibitor, PNP, purine nucleoside phosphorylase, TDF, tenofovir disproxil fumarate, thy, thymidine, TK1, thymidine kinase 1, TTP, thymidine triphosphate, UPLC, ultra high pressure liquid chromatography

## Abstract

Deoxyguanosine kinase (dGK) is reported responsible for the phosphorylation of deoxyadenosine (dA) and deoxyguanosine (dG) in the mitochondrial purine salvage pathway. Antiviral nucleoside analogs known as nucleoside reverse transcriptase inhibitors (NRTIs) must be phosphorylated by host enzymes for the analog to become active. We address the possibility that NRTI purine analogs may be competitive inhibitors of dGK. From a group of such analogs, we demonstrate that entecavir (ETV) competitively inhibited the phosphorylation of dG and dA in rat mitochondria. Mitochondria from the brain, heart, kidney, and liver showed a marked preference for phosphorylation of dG over dA (10–30-fold) and ETV over dA (2.5–4-fold). We found that ETV inhibited the phosphorylation of dG with an IC_50_ of 15.3 ± 2.2 μM and that ETV and dG were both potent inhibitors of dA phosphorylation with IC_50s_ of 0.034 ± 0.007 and 0.028 ± 0.006 μM, respectively. In addition, the phosphorylation of dG and ETV followed Michaelis–Menten kinetics and each competitively inhibited the phosphorylation of the other. We observed that the kinetics of dA phosphorylation were strikingly different from those of dG phosphorylation, with an exponentially lower affinity for dGK and no effect of dA on dG or ETV phosphorylation. Finally, in an isolated heart perfusion model, we demonstrated that dG, dA, and ETV were phosphorylated and dG phosphorylation was inhibited by ETV. Taken together, these data demonstrate that dGK is inhibited by ETV and that the primary role of dGK is in the phosphorylation of dG rather than dA.

Mammals express four deoxynucleoside kinases. Thymidine kinase 1 (TK1) and deoxycytidine kinase (dCK) are both cytoplasmic (1), while thymidine kinase 2 (TK2) and deoxyguanosine kinase (dGK) are mitochondrial kinases. TK1 phosphorylates thymidine (thy) and deoxyuridine and is typically only expressed during S phase of cell division and thus not expressed in nonmitotic cells. The other three deoxynucleoside kinases are thought to be constitutively expressed. dCK phosphorylates deoxycytidine, deoxyadenosine (dA), and deoxyguanosine (dG), while dGK phosphorylates dA, and dG. TK2 phosphorylates thy, deoxycytidine, and deoxyuridine (2, 3, 4). Interestingly, while deficiencies of the cytoplasmic deoxynucleoside kinase have not been associated with disease, the mitochondrial kinases are essential enzymes in the mitochondrial deoxynucleoside salvage pathway and deficiencies of TK2 and dGK are associated with mitochondrial DNA (mtDNA) depletion syndromes (MDSs) that are often severe (5, 6, 7, 8). MDSs are rare autosomal recessive diseases characterized by reduction of mtDNA copy number in specific tissues. Autosomal recessive mutations in the TK2 gene were initially described in 2001 by Saada et al. (9) and cause the myopathic type of MDSs (OMIM # 609560). This type is characterized by childhood onset of muscle weakness associated with mtDNA depletion in skeletal muscle (10). The most severe form has onset in infancy and is rapidly progressive with early death due to respiratory failure. Other forms have a later onset and are more slowly progressive (11, 12). Autosomal recessive mutations of DGUOK (the gene for dGK) causes MDS 3 (hepatocerebral type), OMIM # 251880. This syndrome is characterized by liver failure and neurologic abnormalities in infancy that are progressive, leading to death in the first year of life from liver failure (13). Fourteen percent of MDSs are caused by mutations in the DGUOK gene (14). Patients with dGK deficiency are also reported to have hypoglycemia and lactic acidosis (15). There is also a less severe adult-onset form of dGK deficiency (OMIM # 617070) that is associated with progressive external ophthalmoplegia and recurrent rhabdomyolysis as well as hepatic and neural deficits that present later in infancy or childhood (16). There also appears to be a link between mtDNA depletion and tumorigenesis. Waich *et al* (17) report 6 patients with MDSs harboring biallelic DGUOK mutations, of which 3 are novel, including a large intragenic Austrian founder deletion. One patient aged 6 months was diagnosed with hepatocellular carcinoma. DGUOK was frequently overexpressed in lung adenocarcinoma and aberrant expression of DGUOK correlated with tumor progression and patient overall survival (18). DGUOK may play a role in regulating NAD^+^ biogenesis. Depletion of dGK significantly decreased NAD^+^ level. Furthermore, knockout of the DGUOK considerably reduced expression of the nicotinamide mononucleotide adenylyl transferase, a key molecule controlling NAD^+^ synthesis, at both mRNA and protein levels. Ectopic expression of the nicotinamide mononucleotide adenylyl transferase abrogated the effect of knockdown of DGUOK on NAD^+^ (19).

mtDNA synthesis occurs without any problems during S phase *via* cytosolic supply of dNTPs irrespective of dGK activity, whereas mutations in DGUOK compromise cell cycle–independent mtDNA synthesis (7). Early studies showed that treatment of cells with dGMP and dAMP was capable of partially correcting mtDNA content in DGUOK-mutant cell cultures (7, 8, 20). Later work demonstrated that this improvement was not due directly to dAMP and dGMP but rather to their dephosphorylation to deoxyguanosine and deoxyadenosine, as the monophosphates do not cross the plasma membrane and are quickly dephosphorylated in the culture medium (5, 21). In this study, improvement in mtDNA levels was related to increased cellular levels of deoxyguanosine, as addition of deoxyadenosine had no additional effect. Treatment of a DGUOK knockout in a rat primary hepatocyte model of mtDNA depletion with CERC-913, a dGMP ProTide, worked much better than dG or dGMP treatment (22) as the hydrophobic ProTide masks the phosphate group, increasing stability in the medium and allowing transport into the cell. Once in the cell, the ProTide is cleaved away by esterases releasing dGMP.

Nucleoside reverse transcriptase inhibitors (NRTIs) are nucleoside analog drugs used to slow or stop multiplication of virus-infected cells. The prodrugs are phosphorylated through the salvage pathway in cells to their active forms (1, 23). Previous results from our laboratory showed that 3′-azido-3′-deoxythymidine (AZT), a thymidine analog used to treat HIV patients, was a potent inhibitor of TK2 in the thymidine salvage pathway (24) and decreased the production of thymidine triphosphate (TTP) in isolated mitochondria and in the perfused rat heart, which may account for some of AZT’s toxicity (25, 26). The role of dGK in the phosphorylation of dA and dG and the extent to which various deoxynucleoside purine analogs may interfere with dGK phosphorylation of dG and dA are unknown and are the goals of this study. We used freshly isolated intact functional mitochondria from the liver, heart, brain, and kidney to investigate the activity and kinetics of dGK and its role in phosphorylating dA and dG. We chose to use isolated functioning intact mitochondria for this study rather than purified enzymes to provide data that are more relevant to the natural setting of mitochondria that includes intact membranes and transport systems, as well as active metabolic processes of energy generation and biosynthesis. With this approach, we can deduce potential tissue differences observed in the organelle setting that cannot be obtained using purified enzymes. The effects of various common deoxynucleoside purine analogs, including entecavir (ETV), adefovir, tenofovir, tenofovir disproxil fumarate (TDF), lobucavir, penciclovir, ganciclovir, and dideoxyguanosine, on dA and dG phosphorylation were determined. Of those analogs tested, only ETV was found to inhibit the phosphorylation of dG and dA. Additional results will show that while dGK phosphorylates both dG and dA, it demonstrates a marked preference for dG (1, 4, 27). Further, we show for the first time that dG was a potent inhibitor of the phosphorylation of dA, while dA had no effect on the phosphorylation of dG.

As ETV was inhibitory, we selected it for additional study. ETV is a drug used in the treatment of chronic hepatitis B and is a deoxyguanosine analog (23, 28, 29). While ETV is generally considered to be safe and well tolerated (30), there are reports of severe and fatal lactic acidosis related to mitochondrial toxicity on long-term treatment (31, 32). As it has been reported that ETV-triphosphate is not recognized by mitochondrial polymerase γ, this common target of mitochondrial toxicity is unlikely to account for toxicity of ETV (33). Rather, toxicity if observed may be caused by competition with dA and dG for phosphorylation, decreasing dATP or dGTP levels as noted above for AZT inhibition of thymidine phosphorylation.

ETV has been shown to be readily phosphorylated in hepatocyte cell culture; however, the deoxykinase(s) involved have never been investigated (34). Our results will show for the first time that ETV is readily phosphorylated in isolated mitochondria, presumably by dGK and, as noted earlier, inhibited the phosphorylation of dA and dG, but was much more potent in inhibiting dA. Thus, ETV has the potential to inhibit the purine deoxynucleoside salvage pathway and reduce the level of deoxypurine nucleotides, leading to a potential imbalance in dNTP pools which has been associated with a decrease in mitochondrial DNA copy number (5, 9).

## Results

### Pilot study of the effect of dG and dA nucleoside analogs on dA or dG phosphorylation

**Figure 1:**
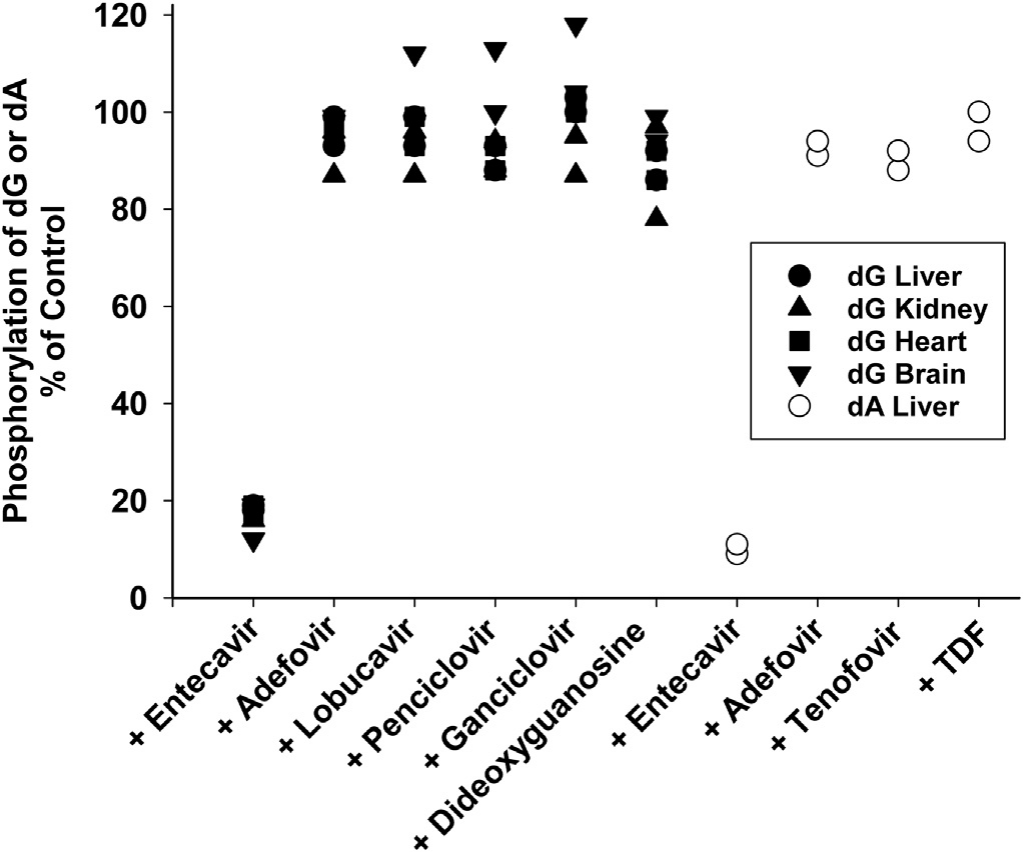
**Amount of [**^**3**^**H]-dA or[**^**3**^**H]-dG phosphorylation in the presence 100 μM dA or dG analogs.** [^3^H]-dG (0.5 μM) (3000 DPM/pmol) was incubated in isolated liver, kidney, heart, and brain mitochondria in the presence or absence of 100 μM dG analogs for 120 min 0. [^3^H]-dA (5 μM) (3000 DPM/pmol) was incubated with liver mitochondria only. Immucillin-H (I-H) (2 μM) and EHNA (5 μM) were added to the incubation mixture to prevent the breakdown of substrate by purine nucleoside phosphorylase (PNP) and adenosine deaminase (ADA) activity, respectively. The results were plotted as the sum of all three phosphorylated forms (monophosphate ∼20%, diphosphate ∼5%, and triphosphate ∼75% for both precursors), expressed as a percent of phosphorylation in the control sample for each tissue mitochondria. The results for [^3^H]-dG phosphorylation are individual points from four different tissues. Results with [^3^H]-dA are individual points from liver mitochondria only. dA, deoxyadenosine; dG, deoxyguanosine; EHNA, erythro-9-Amino-β-hexyl--α-methyl-9H-purine-9-ethanol hydrochloride, erythro-9-(2-Hydroxy-3-nonyl)-adenine hydrochloride; TDF, tenofovir disproxil fumarate.

dG and dA analogs including, ETV, adefovir, tenofovir, TDF, lobucavir, penciclovir, ganciclovir, and dideoxyguanosine were incubated at high concentration (100 μM) with respect to their natural analogs. [^3^H]-dG (0.5 μM) was incubated in isolated liver, kidney, heart, and brain mitochondria, while [^3^H]-dA (0.5 μM) was incubated only in liver mitochondria for 2 h in the presence of erythro-9-Amino-β-hexyl--α-methyl-9H-purine-9-ethanol hydrochloride, erythro-9-(2-Hydroxy-3-nonyl)-adenine hydrochloride (EHNA) (5 μM) and Immucillin H (I-H) (2.0 μM) as described in the Experimental procedures (Fig. 1). ETV was shown to be the only dG/dA analog of the eight tested that showed significant inhibition of the phosphorylation of dA and dG. As a result, ETV was chosen for further study.

### Tissue-specific phosphorylation of [^3^H]-dG, [^3^H]-dA, and [^3^H]-ETV

**Figure 2:**
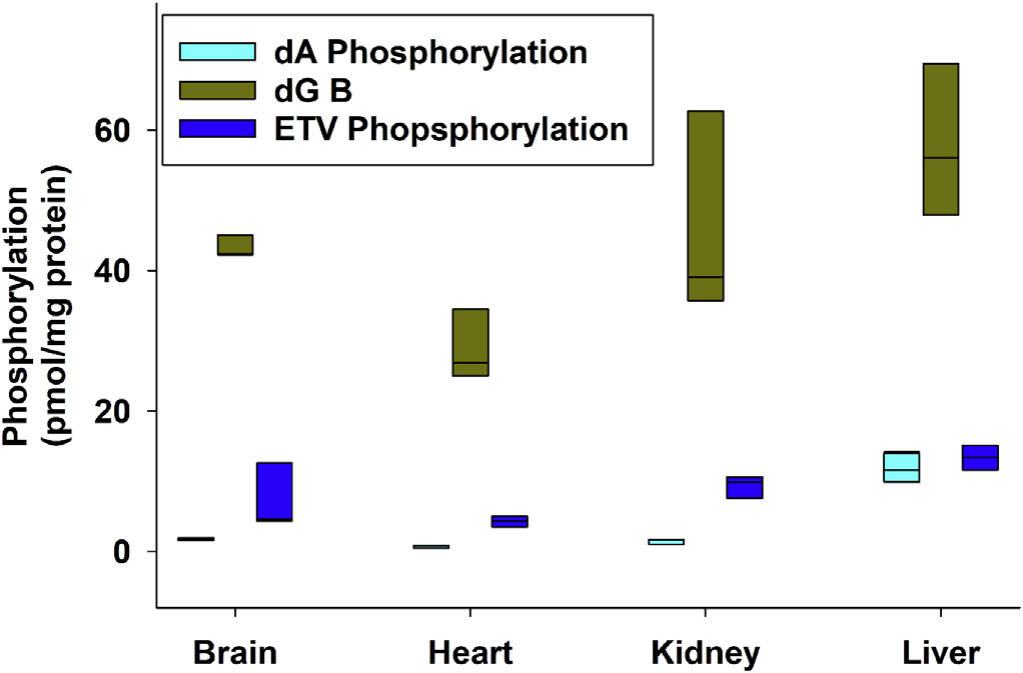
**Phosphorylation rates of [**^**3**^**H]-dA, [**^**3**^**H]-dG, or [**^**3**^**H]-ETV in mitochondria from the brain, heart, kidney, and liver.** Mitochondria were isolated from the rat brain, heart, kidney, and liver and were incubated with 1 μM of [^3^H]-dA, [^3^H]-dG, or [^3^H]-ETV all at ∼3000 DPM/pmol for 2 h as described for Figure 1 and the results plotted as pmol phosphorylated product/mg protein for mitochondria from each tissue. The data are shown as a *box plot* of 3 independent observations. dA, deoxyadenosine; dG, deoxyguanosine; ETV, entecavir.

To detect potential tissue-specific effects, we studied the phosphorylation of [^3^H]-dG, [^3^H]-dA, or [^3^H]-ETV, all at 1 μM concentrations, in mitochondria freshly isolated from four postmitotic tissues, heart, liver, kidney, and brain. All four preparations of tissue mitochondria produced monophosphates (∼20%), diphosphates (∼< 5%), and triphosphates (∼75%) in the relative amounts shown. As noted in the Experimental procedures, these were added together to determine total phosphorylation. As shown in Figure 2, dG was phosphorylated about 10- to 30-fold more readily than dA and 5- to 7-fold more readily than ETV. With the exception of liver mitochondria, ETV was phosphorylated about 2.5 to 4 times better than dA. Phosphorylation of dA was the lowest in all four mitochondrial preparations and was barely detectable in the heart. The liver had the highest rate of phosphorylation of all three precursors, followed by the kidney, brain, and heart (Fig. 2). Phosphorylation of [^3^H]-dG was sufficiently robust to measure in mitochondria isolated from any of the four tissues; however, further experiments on [^3^H]-dA phosphorylation were done only in isolated liver mitochondria.

### Effects of dA, dG, and ETV concentrations on phosphorylation of each other

**Figure 3:**
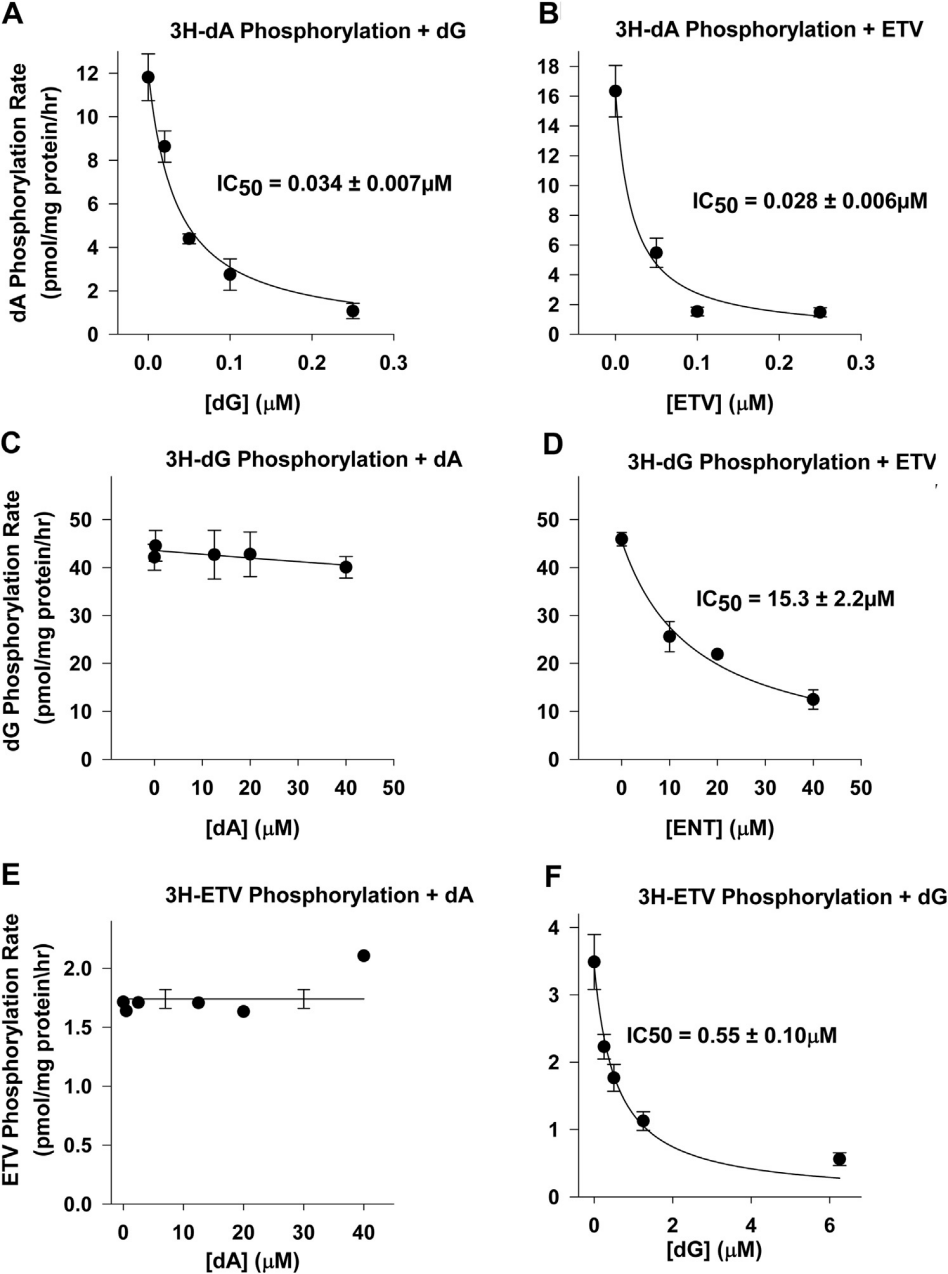
**Effect of competing deoxynucleosides on the phosphorylation rates of [**^**3**^**H]-dA, [**^**3**^**H]-dG, or [**^**3**^**H]-ETV.** Mitochondria were incubated for 60 min as described for Figure 1 and the total amount of phosphorylation calculated as described for Figure 1. *A*, phosphorylation rate of 1 μM [^3^H]-dA, ∼3000 DPM/pmol, in liver mitochondria in the presence of dG (0–0.25 μM). *B*, phosphorylation rate of 1 μM [^3^H]-dA, 3000 DPM/pmol, in liver mitochondria in the presence of ETV (0–0.25 μM). *C*, phosphorylation rate of 1 μM [^3^H]-dG, 3000 DPM/pmol, in heart mitochondria in the presence of dA (up to 40 μM). *D*, phosphorylation rate of 1 μM [^3^H]-dG, 3000 DPM/pmol, in heart mitochondria in the presence of ETV (0–40 μM). *E*, phosphorylation rate of 1 μM [^3^H]-ETV, 3000 DPM/pmol, in liver mitochondria in the presence of dA (0–40 μM). *F*, phosphorylation rate of 1 μM [^3^H]-ETV, 3000 DPM/pmol, in liver mitochondria in the presence of dG (0–6.5 μM). For all panels, the best-fit rate of incorporation over 60 min was plotted against inhibitor concentration. The IC_50_s were calculated from the best-fit hyperbolic decay line (y = ab/(b + x) by SigmaPlot 14.0. The data shown (except (*E*)) are the mean and SEM of phosphorylation in 3 independent observations. As the rate shown in (E) was essentially the same for all dA concentrations, the experiment was not repeated and the values were averaged (line) ± standard error of the mean. dA, deoxyadenosine; dG, deoxyguanosine; ETV, entecavir.

As dG, dA, and ETV are all similar compounds, it might be expected that they competitively inhibit each other’s phosphorylation. To compare the relative extent of this inhibition, we determined IC_50_ curves for each labeled compound in the presence of the unlabeled competing compound. To make comparisons between compounds, it was necessary to use the same concentration for each labeled compound. Cellular levels of dG and dA to our knowledge have never been reported, presumably due to the difficulty in measuring these in the face of much higher levels of their ribonucleosides. In our hands, the levels of dA and dG in rat tissues were below the limits of detection, < 5 μM. As plasma levels of deoxycytidine and deoxyuridine have been reported at about 1 μM (22), we have chosen to use 1 μM as a physiological estimate of dA, dG, and ETV to compare the relative IC_50_ inhibition of each of these compounds in the presence of each other (Fig. 3). As shown, in Figure 3, 1 μM of [^3^H]-dA phosphorylation in liver mitochondria was potently inhibited by dG (Fig. 3*A*) or ETV, a guanosine analog (Fig. 3*B*), with very similar IC_50_ values of 0.034 ± 0.007 and 0.028 ± 0.006 μM, respectively. In contrast, [^3^H]-dG phosphorylation in heart mitochondria was not inhibited at all by the presence of up to 40 μM dA (Fig. 3*A*). ETV also inhibited [^3^H]-dG phosphorylation, but with a much higher IC_50_ value of 15.3 ± 2.2 μM (Fig. 3*I*). The IC_50_ of ETV inhibition of [^3^H]-dG phosphorylation in mitochondria from the brain, kidney, or liver (18.9 ± 6.2, 14.3 ± 3.3, and 15.7 ± 1.8 μM), respectively) were quite similar. In contrast, the inhibition of [^3^H]-ETV phosphorylation in liver mitochondria by dG yielded a much more potent IC_50_ of 0.55 ± 0.10 μM (Fig. 3*F*), indicating the preference of dGK for its natural substrate, dG over ETV. As above for dG, [^3^H]-ETV phosphorylation in liver mitochondria was not inhibited at all by the presence of up to 40 μM dA (Fig. 3*E*).

### Kinetic properties of [^3^H]-dG and [^3^H]-ETV phosphorylation and cross inhibition

**Figure 4:**
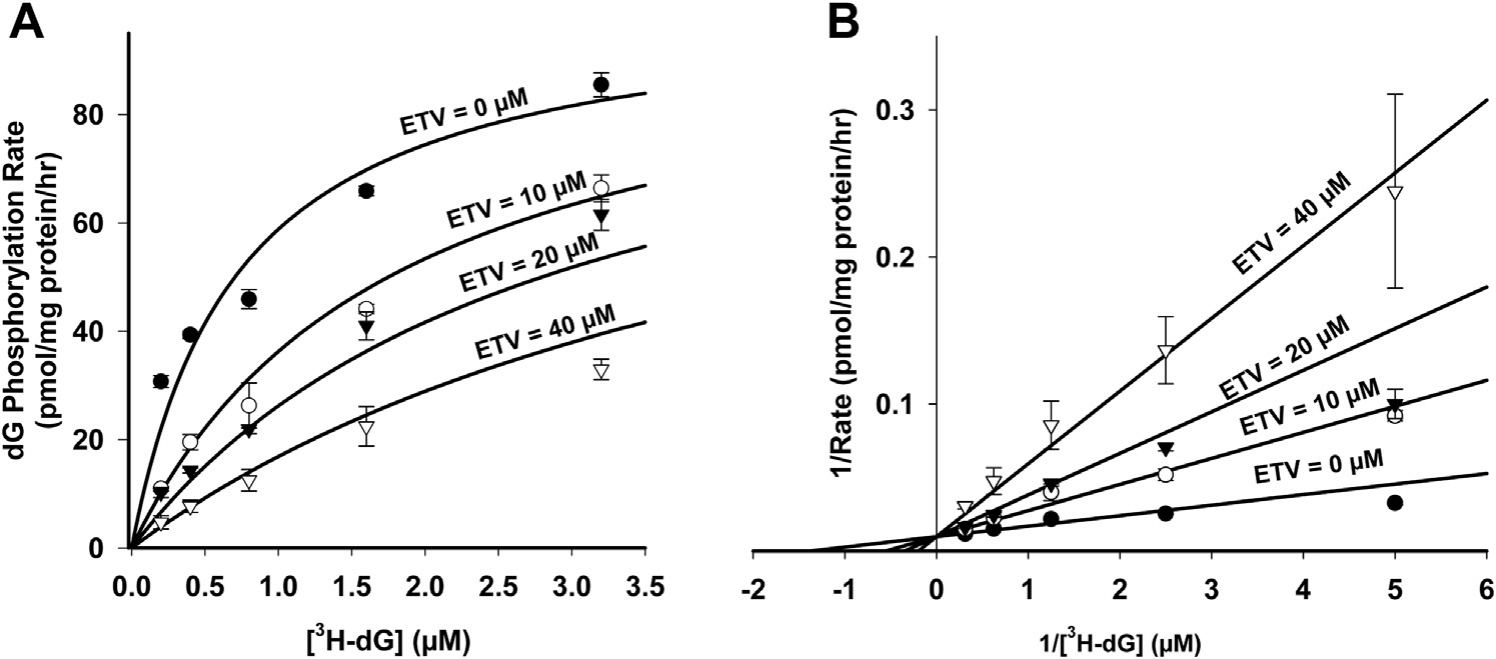
**Kinetic analysis of phosphorylation rate of [**^**3**^**H]-dG in the presence of ETV.** Mitochondria were incubated for 60 min as described for Figure 1 and the total amount of phosphorylation calculated as described for Figure 1. *A*, Michaelis–Menten plot of [^3^H]-dG phosphorylation in the presence of 0, 10, 20, and 40 μM ETV. The best-fit rate of incorporation over 60 min was plotted against substrate concentration. *B*, Lineweaver–Burk reciprocal plot of data show in (*A*) and described in the Experimental procedures. The best fit of the data by the SigmaPlot 14.0 kinetic program is shown and demonstrates that ETV is a competitive inhibitor of dG phosphorylation. The data shown are the mean and SEM of 3 independent studies. See text for further details. dG, deoxyguanosine; ETV, entecavir.

**Figure 5:**
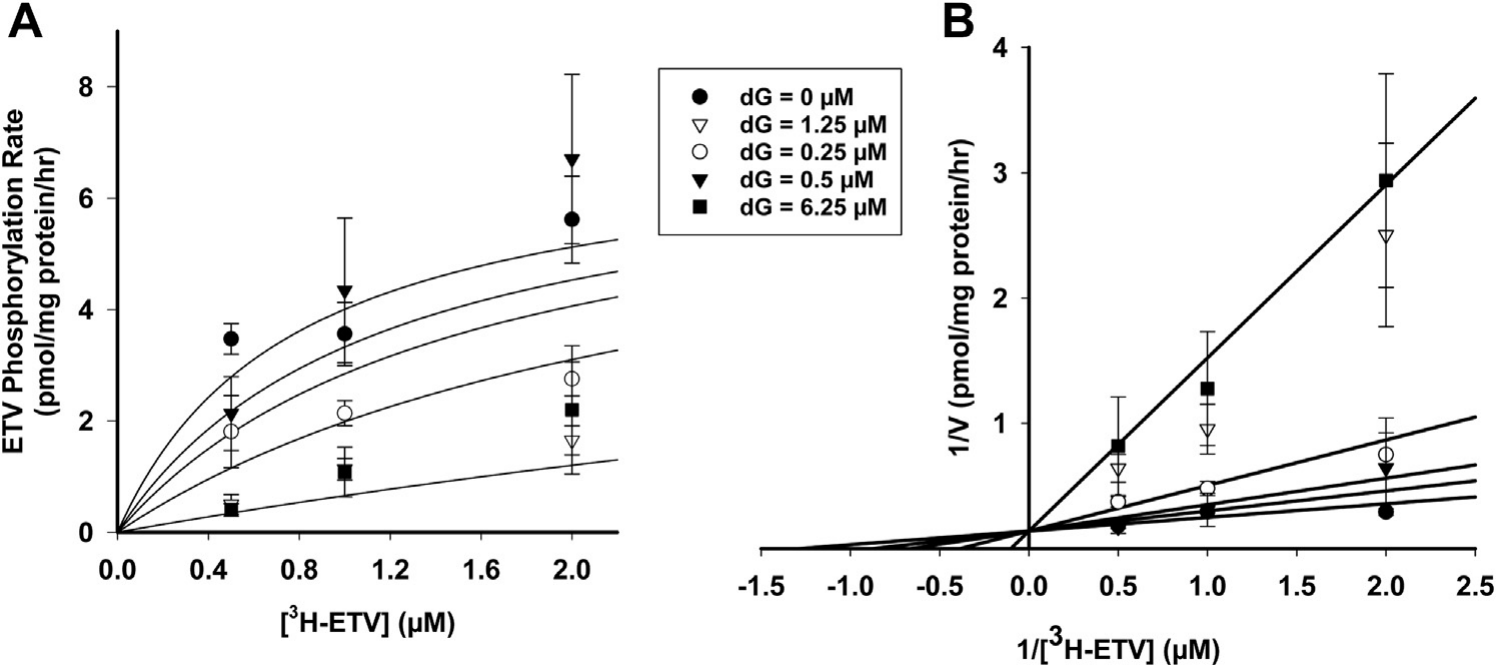
**Kinetic analysis of phosphorylation rate of [**^**3**^**H]-ETV in the presence of dG.** Mitochondria were incubated for 60 min as described for Figure 1, and the total amount of phosphorylation calculated as described for Figure 1. *A*, Michaelis–Menten plot of [^3^H]-ETV phosphorylation in the presence of 0, 0.25, 0.5, 1.25, and 6.25 μM dG, in heart mitochondria. The best-fit rate of incorporation over 60 min was plotted against substrate concentration. *B*, Lineweaver–Burk reciprocal plot of data shown in (*A*) and described in the Experimental procedures. The best fit of the data by the SigmaPlot 14.0 kinetic program is shown and demonstrates that dG is a competitive inhibitor of ETV phosphorylation. The data shown are the mean and SEM of 3 independent studies. See text for further details. dG, deoxyguanosine; ETV, entecavir.

The kinetic properties of [^3^H]-dG and [^3^H]-ETV phosphorylation were studied in freshly isolated heart mitochondria. The K_m_ values of dG and ETV were 0.72 ± 0.09 μM (Fig. 4*B*) and 0.77 ± 0.62 μM (Fig. 5*B*), with accompanying V_max_ values of 101 ± 5 pmol/mg protein/hr (Fig. 4*B*) and 7.1 ± 2.2 pmol/mg protein/hr (Fig. 5*B*), respectively. These data indicate that dG and ETV bind with similar affinity to the enzyme but that phosphorylation of ETV is far less efficient than phosphorylation of dG. The effect of ETV on [^3^H]-dG phosphorylation was determined at four inhibitor concentrations. The rates obtained were analyzed for the best fit to three models of inhibition, competitive, noncompetitive, and uncompetitive using the kinetic wizard in Sigma Plot 14.0. As shown in Figure 4*B*, the best fit was obtained using a competitive model of inhibition with a K_i_ of 6.7 ± 0.8 μM. Given the similarity of structure between ETV and dG, competitive inhibition was expected. In turn, the effect of dG on [^3^H]-ETV phosphorylation at four different inhibitor concentrations was similarly determined as shown in Figure 5. As with ETV inhibition of dG phosphorylation, the inhibition of ETV phosphorylation by dG was also competitive with a much more potent K_i_ of 0.53 ± 0.38 μM.

### Effect of dG and ETV on kinetic properties of [^3^H]-dA phosphorylation

**Figure 6:**
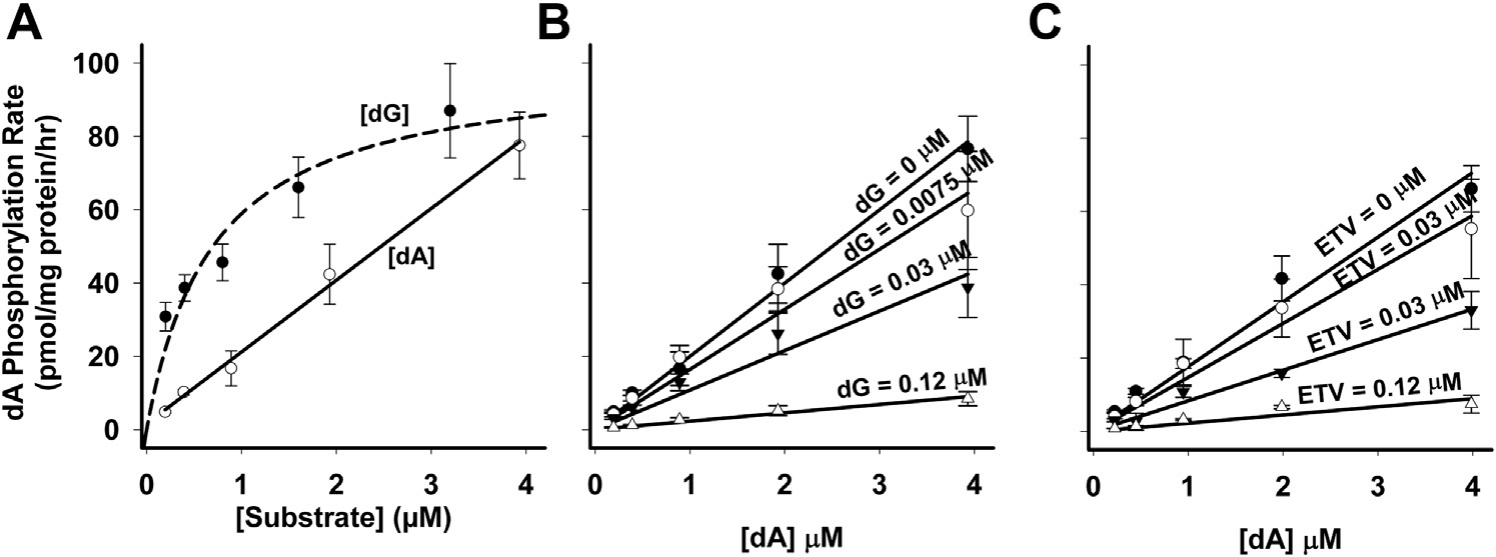
**Kinetic analysis of [3H]-dA phosphorylation in the presence of dG or ETV in isolated liver mitochondria.** Mitochondria were incubated for 60 min as described for Figure 1, and the total amount of phosphorylation calculated as described for Figure 1. *A*, comparison of the rate of [^3^H]-dA phosphorylation (solid line) plotted against substrate concentration to the rate of [^3^H]-dG phosphorylation (dashed line, from Fig. 4*A*). As [^3^H]-dA phosphorylation was linear at the concentrations used, it could not fit a Michaelis–Menten curve. [^3^H]-dA appears to have a much higher K_m_ and V_max_ than [^3^H]-dG, but the values cannot be obtained from these data. *B*, linear regression plot of [^3^H]-dA phosphorylation in the presence of 0, 0.0075, 0.03, and 0.12 μM dG, as described in the Experimental procedures. *C*, linear regression plot of [^3^H]-dA phosphorylation in the presence of 0, 0.0075, 0.03, and 0.12 μM ETV as described in the Experimental procedures. The data shown are the mean and SEM of 3 independent studies. See text for more details. dA, deoxyadenosine; dG, deoxyguanosine; ETV, entecavir.

As [^3^H]-dA was poorly phosphorylated in heart mitochondria, the phosphorylation of [^3^H]-dA was studied in the more robust liver mitochondrial preparation. Our initial experiments (Fig. 6*A*) clearly showed a major difference in phosphorylation of dA *versus* dG in that dA has an exponentially lower affinity for the enzyme such that up to 4 μM [dA], phosphorylation is still exhibiting linear kinetics. Thus, it was not possible from this experiment to establish a K_m_ or V_max_ for dA phosphorylation without going to significantly higher concentrations than would ever be expected *in vivo*. However, it was still quite clear that dG (Fig. 6*B*) and ETV (Fig. 6*C*) potently inhibited dA phosphorylation and that it is likely to represent competitive inhibition. Note that a dG concentration of 0.12 μΜ nearly completely inhibited the phosphorylation of the highest concentration of dA studied (4 μM). These data strongly suggest that the enzyme dGK functions under most conditions to phosphorylate dG rather than dA.

### Phosphorylation of [^3^H]-GMP and [^3^H]-AMP in isolated heart mitochondria

**Figure 7:**
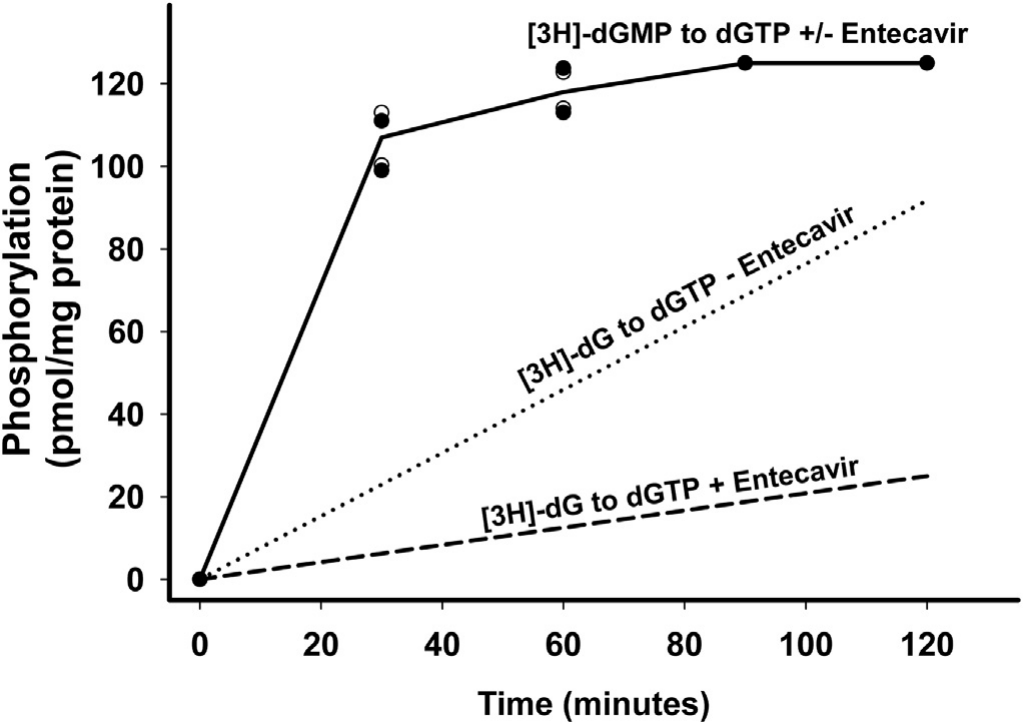
**Comparison of phosphorylation rates of dGMP *versus* dG in isolated heart mitochondria.** [^3^H]-dGMP (1 μM) was incubated in heart mitochondria as described for Figure 1 for the times shown and conversion to [^3^H]-dGTP was determined in the presence and absence of ETV (40 μm), shown earlier as a competitive inhibitor of dGK. The data shown for [^3^H]-dGMP in the presence and absence of ETV are the average of two experiments, both of which yielded identical curves in the presence and absence of ETV. The plateau data (123 ± 1 pmol/mg protein) represent complete conversion of [^3^H]-dGMP to [^3^H]-dGTP. The data for [^3^H]-dG phosphorylation to [^3^H]-dGTP in the presence (*dotted line*) and absence of ETV (dashed line) were taken from the rate data for the control (mean ± SEM = 45.9 ± 1.8 pmol/mg protein/hr) and 40 μM ETV (mean and SEM = 12.4 ± 2.0 pmol/mg protein/hr) data of Figure 4 and shown here for comparison. dG, deoxyguanosine; dGK, deoxyguanosine kinase; ETV, entecavir.

**Figure 8:**
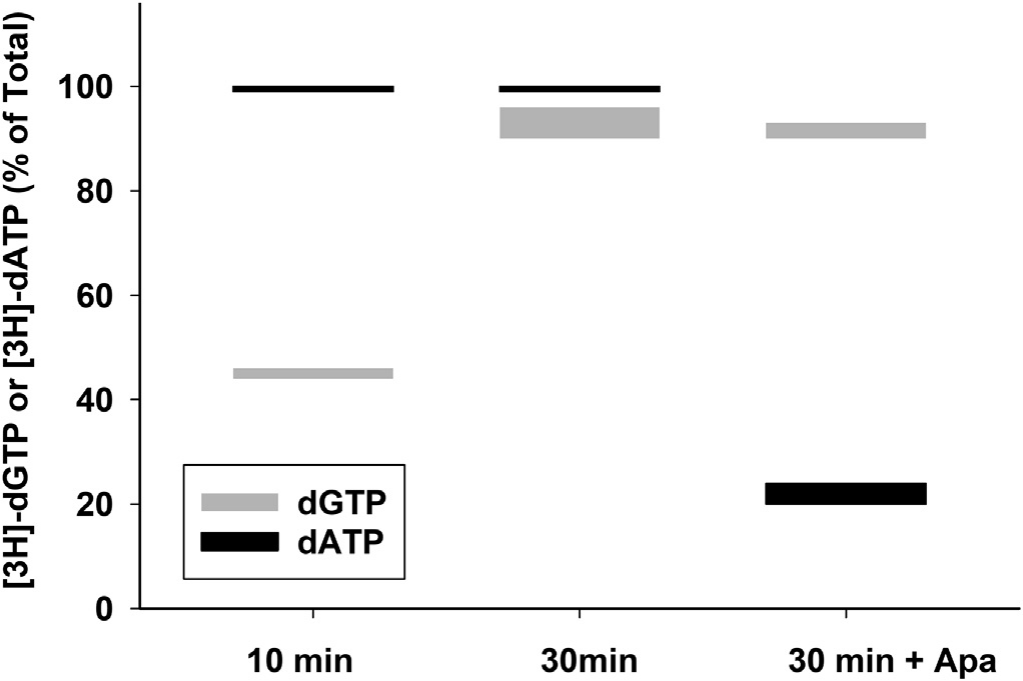
**Phosphorylation rates of [**^**3**^**H]-dAMP compared to [**^**3**^**H]-dGMP in the presence and absence of Ap5A in isolated heart mitochondria.** [^3^H]-dGMP (1 μM) or [^3^H]-dAMP (1 μM) was incubated in heart mitochondria as described for Figure 1 for the times shown, and their conversion to [^3^H]-dGTP and [^3^H]-dATP, respectively, determined in the presence and absence of Ap5A (40 μm), a competitive inhibitor of adenylate kinase. Data are shown as a box plot of two independent experiments. The thickness of the lines indicates the spread of the duplicates. Ap5A, P1,P5-di(adenosine-5) pentaphosphate pentasodium salt.

In previous experiments studying thymidine phosphorylation in isolated mitochondria, we demonstrated that TMP could only be converted to TTP by first being dephosphorylated to thymidine (24). Thus, we were interested in determining if the dGTP and dATP phosphorylation pathway in isolated mitochondria functioned similarly. As shown in Figure 7, [^3^H]-dGMP (1 μM) was quantitatively converted to [^3^H]-dGTP in heart mitochondria within 40 to 60 min of incubation. At the 30-min time point, the conversion of [^3^H]-dGMP to [^3^H]-dGTP (106 ± 4 pmol/mg protein) compared to the conversion of [^3^H]-dG to [^3^H]-dGTP (22.4 ± 0.8) was 4.6 times faster (*p* < 0.001). ETV had no effect on this pathway, indicating that dephosphorylation of dGMP to dG was not necessary and that the phosphorylation of dG is clearly a rate-limiting step in the phosphorylation pathway of dGTP. As shown in Figure 8, [^3^H]-dAMP in heart mitochondria was completely converted to [^3^H]-dATP within 10 min, far faster and more complete than the phosphorylation of [^3^H]-dA. The conversion was inhibited by P1,P5-di(adenosine-5) pentaphosphate pentasodium salt (Ap5A), an inhibitor of adenylate kinase, demonstrating that dAMP is a good substrate for this rapidly acting equilibrative enzyme. The phosphorylation of dGMP was not inhibited by Ap5A, indicating that the phosphorylation of dGMP was not catalyzed by adenylate kinase. Identical results for both [^3^H]-dAMP and [^3^H]-dGMP were obtained in liver mitochondria.

### [^3^H]-dG, [^3^H]-dA, and [^3^H]-ETV stability and phosphorylation in isolated rat heart perfusion

**Figure 9:**
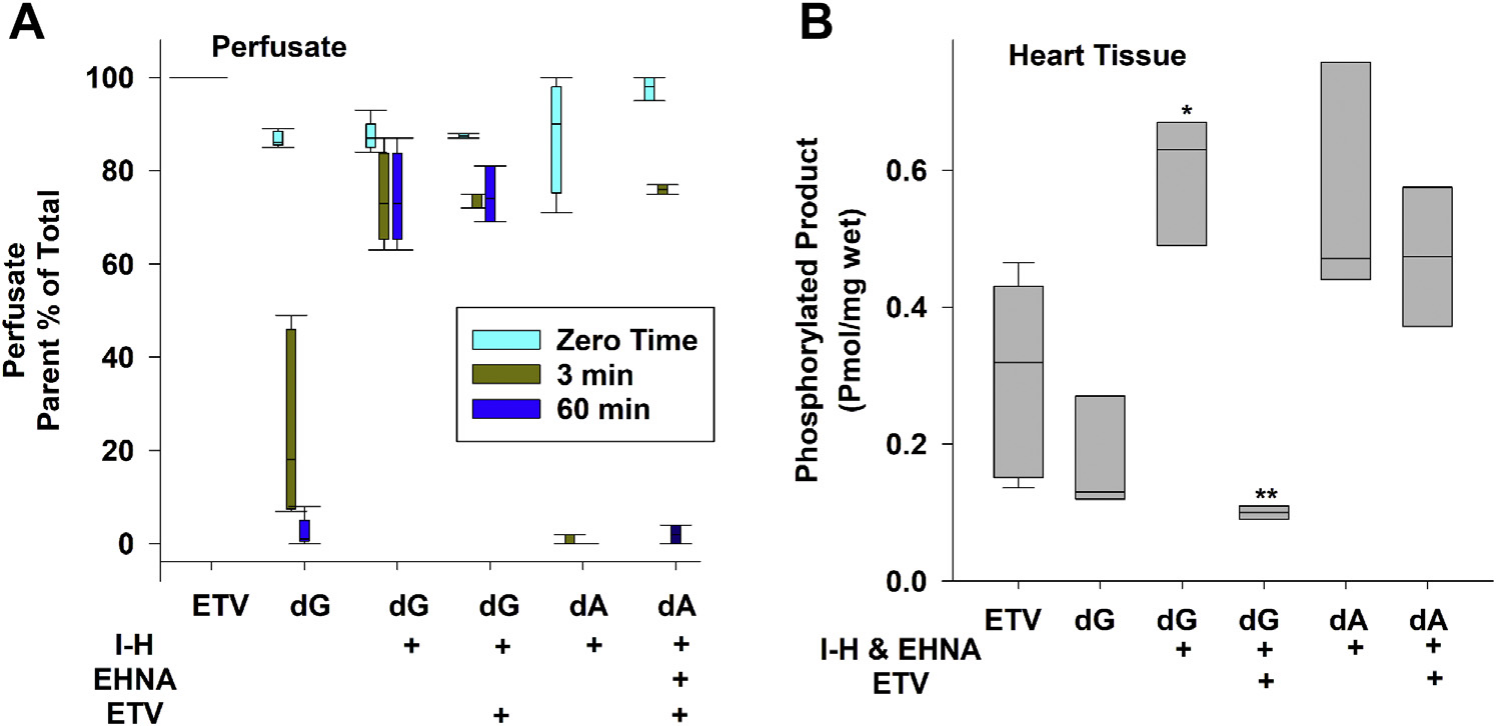
**[**^**3**^**H]-ETV and [**^**3**^**H]-dG phosphorylation in a perfused rat heart.** Rat hearts were removed and perfused using the Langendorff method for 1 h as described in Experimental procedures with 0.5 μM [^3^H]-ETV, 0.5 μM [^3^H]-dG), or 0.5 μM [^3^H]-dA. Immucillin-H (+I-H) (2 μM), 5 μM EHNA, and/or 100 μM ETV (ETV) were added as indicated. *A*, the amount of exogenous [^3^H]-parent compound in the perfusate as a function of time, plotted as a box plot of three independent experiments. See text for details. *B*, the amount of phosphorylated [^3^H]-ETV, [^3^H]-dG, or [^3^H]-dA in the heart tissue after 60 min of perfusion with additions as noted in (*A*). See text for details. The data are shown as a box plot of 3 to 5 independent experiments. ∗ = *p* < 0.005 for dG + I-H and EHNA compared to dG alone. ∗∗ = *p* < 0.0004 for dG + I-H and EHNA and ETV compared to dG + I-H and EHNA. dA, deoxyadenosine; dG, deoxyguanosine; ETV, entecavir; EHNA, erythro-9-Amino-β-hexyl--α-methyl-9H-purine-9-ethanol hydrochloride, erythro-9-(2-Hydroxy-3-nonyl)-adenine hydrochloride.

The phosphorylation of [^3^H]-dG, [^3^H]-dA, and [^3^H]-ETV in isolated mitochondria depends on the activity of the mitochondrial dGK. To determine the degree of phosphorylation in a whole tissue, which would include the activity of the cytosolic dCK, we utilized an isolated rat heart Langendorff retrograde perfusion model. The breakdown and phosphorylation of [^3^H]-ETV, [^3^H]-dG, and [^3^H]-dA (0.5 μM each) was measured in the perfusates and in the hearts after 60 min of perfusion (Fig. 9, *A* and *B*). [^3^H]-ETV was completely resistant to breakdown during perfusion (Fig. 9, Panel *A*) and generated 0.25 ± 0.05 pmol/mg wet heart phosphorylated product (Fig. 9, Panel *B*) during the 60-min perfusion. Conversely, [^3^H]-dG was broken down rapidly in the perfusate from an initial level of 86.8 ± 0.7% to 25 ± 8.8% within just 3 min and was nearly completely converted to catabolites by 60 min of perfusion. However, the heart was still able to take up and maintain a small amount of dG to generate 0.18 ± 0.05 pmol/mg wet heart of phosphorylated dG during the 60-min perfusion. The addition of I-H (2 μM), which inhibits purine nucleoside phosphorylase (PNP), prevented most of the breakdown of dG in the perfusate (Fig. 9*A* + I-H), resulting in a three-fold increase in phosphorylated [^3^H]-dG to 0.57 ± 0.08 pmol/mg wet heart compared to [^3^H]-dG alone (Fig. 9*B*, *p* < 0.005). Finally, adding unlabeled ETV (100 μM) to [^3^H]-dG + I-H inhibited [^3^H]-dG phosphorylation by 82.0 ± 0.7%, *p* < 0.0004 (Fig. 9*B*). [^3^H]-dA was even more susceptible to catabolism during perfusion, with the parent compound completely lost by 3 min even in the presence of I-H (Fig. 9*A*). The addition of EHNA to the perfusate provided only modest protection, as only 47.5% of the [^3^H]-dA remained by 3 min with complete loss by 60 min (Fig. 9*A*). Despite the loss of [^3^H]-dA from the perfusate, the heart took up and maintained enough dA to produce 0.48 ± 0.08 pmol/mg wet heart of phosphorylated [^3^H]-dA products at 60 min (Fig. 9*B*). This was similar to the amount of phosphorylated [^3^H]-dG observed in the presence of I-H. However, the further addition of ETV to the perfusate did not appear to inhibit [^3^H]-dA phosphorylation (Fig. 9*B*), suggesting that [^3^H]-dA phosphorylation in the perfused heart was not carried out by dGK, and perhaps was a function of dCK. Note that [^3^H]-dG and [^3^H]-dG catabolites equilibrate rapidly between the perfusion buffer and the heart, while the phosphorylated forms of [^3^H]-dG are retained solely in the heart tissue.

## Discussion

In previous studies performed by our laboratory, we demonstrated that the NRTI thymidine analog AZT inhibited the ability of TK2 to phosphorylate thymidine in postmitotic tissues leading to decreased TTP levels and unbalanced dNTP pools (24, 25, 26). As unbalanced dNTP pools have been shown to cause mtDNA depletion (35) and TK2 deficiencies lead to severe mtDNA depletion (10), we hypothesized this may account for the observed toxicity in heart upon AZT treatment (36). As all NRTIs use a salvage pathway for their activation, it might be expected that other NRTIs may have similar competitive inhibitory effects on the phosphorylation of the natural deoxynucleoside substrates and similar mitochondrial toxicity (37, 38). To uncover this potential, seven common dA and dG analogs were tested in isolated liver mitochondria to determine their potential to inhibit the dA/dG phosphorylation pathway in mitochondria (Fig. 1). Of these seven, ETV was the only one with inhibitory properties, and as such, the effects of ETV on the phosphorylation of dA and dG were characterized further in mitochondria isolated from the heart, liver, brain, and kidney. While ETV is generally considered to be safe and well tolerated (30), there are reports of severe and fatal lactic acidosis related to mitochondrial toxicity on long-term treatment (31, 32). As it has been reported that ETV-triphosphate is not recognized by mitochondrial polymerase γ, this common mechanism of NRTI toxicity is unlikely to account for toxicity of ETV (33). Rather, toxicity if observed may be caused by competition with dA and dG for phosphorylation, as noted for AZT inhibition of thymidine phosphorylation associated with mitochondrial DNA depletion. Interestingly, one report demonstrated that peripheral blood mononuclear cells isolated from patients treated for 2 to 5 years with ETV had significantly reduced levels of mtDNA compared to nuclear DNA (39), although clinical manifestations were not noted. The lack of clinical manifestations may be related to well-known threshold effects of mitochondrial function, and severe toxicity may only be noted in patients that have other underlying mitochondrial problems.

While the deoxynucleosides dA, dG, and ETV were all phosphorylated, the rates between tissues and between deoxynucleosides varied considerably (Fig. 2). ETV was phosphorylated about 15 to 20% of the rate of dG phosphorylation, but 2.5 to 4 times better than dA phosphorylation. While the phosphorylation of ETV in cultured cells (34) has been reported, the kinase responsible for that phosphorylation was not identified. To our knowledge, this is the first time it has been shown that ETV can be phosphorylated specifically by isolated mitochondria, presumably by mitochondrial dGK. In earlier work (40), we demonstrated that the mitochondria isolated by our technique are well washed and free of cytosolic contaminants; thus, this phosphorylation is unlikely to be mediated by the cytosolic dCK enzyme.

dG phosphorylation was 10 times faster than dA phosphorylation in mitochondria from all four tissues (Fig. 2). This is consistent with the report from Johansson *et al*., which isolated purified dGK much preferred dG as a substrate over dA (4, 27). Liver mitochondria had the highest overall activity, converting 60 pmol/mg mitochondrial protein of dG to phosphorylated products, while mitochondria from the heart had the lowest activity, converting 30 pmol/mg mitochondrial protein of dG to phosphorylated products, with phosphorylation of dA being barely detectable (Fig. 2). This differs from the activity thymidine kinase 2, which was most active in mitochondria from the brain and kidney, followed by mitochondria from the liver and heart (manuscript in preparation). The reasons for these tissue-specific differences in activity are unclear. This may reflect the level of expression of these proteins per milligram of mitochondrial protein in mitochondria from each tissue. Alternatively, the difference could reflect other factors regulating the phosphorylation reaction. The level of mRNA for dGK in the heart (41) and liver (unpublished data) is essentially the same. Future work will use Western blots to determine protein levels in the different tissue mitochondria.

The data in Figures 3, 4 and 5 clearly demonstrated that dG and ETV competitively inhibited the phosphorylation of each other. At 1 μM physiological concentration, the IC_50_ inhibition of dG phosphorylation by ETV was 15.3 ± 2.2 μM (Fig. 3*D*), while the inhibition of ETV phosphorylation by dG was 0.25 ± 0.18 μM, clearly indicating the preference of dGK for its natural dG substrate. The C_max_ of ETV in treatment is reported to be 4.2 ng/ml (0.015 μM) at a 0.5-mg/day dose and 8.2 ng/ml (0.027 μM) for a 1-mg/day dose. This is 560-fold below the IC_50_ of ETV on dG phosphorylation, and unless the tissue concentration of dG is well below 0.1 uM, it is unlikely that ETV ever reaches a level that would inhibit dG phosphorylation by dGK in otherwise normal treated individuals (30). However, both dG and ETV were potent inhibitors of dA phosphorylation with IC_50_ values at 1 μM dA (Fig. 3*A*) of 0.034 ± 0.007 and 0.028 ± 0.006 μM, respectively (Fig. 3*B*), 500-fold more potent than ETV inhibition of dG phosphorylation. The potent inhibition of dA phosphorylation at treatment levels of ETV suggests that ETV could decrease the synthesis of dATP, particularly in postmitotic cells that downregulate the *de novo* deoxynucleoside synthesis pathways. Thus, ETV could result in mitochondrial DNA depletion and mitochondrial toxicity, as noted earlier.

The data in Figures 4 and 5 demonstrate that the phosphorylation of dG and ETV, respectively, follow Michaelis–Menten kinetics and competitively inhibit each other with a K_i_ of 6.7 ± 0.8 μM for ETV inhibition of dG phosphorylation and a K_i_ of 0.53 ± 0.38 μM for dG inhibition of ETV phosphorylation. However, the data for dA in Figure 6 demonstrate a striking difference in the kinetics of phosphorylation of dA *versus* dG, with dA displaying an exponentially lower affinity for the enzyme. This led to a kinetic plot for dA phosphorylation that was linear up to 4 μM dA. This is consistent with the results of Sjoberg *et al* on recombinant purified dGK (4) and accounts for the lack of effect of dA on dG and ETV phosphorylation in Figure 3, *C* and *E*. As shown in Figures 4 and 6, dG is a potent inhibitor of dA phosphorylation, with 0.12 μM dG nearly completely inhibiting dA phosphorylation at 4 μM (Fig. 6). While the level of dG in mitochondria has not been reported, dGTP is reported to represent >90% of the deoxynucleoside triphosphate pool in heart and liver mitochondria (42). This pool is likely to give rise to some dG during isolated mitochondrial incubations and may provide an endogenous pool of dG that might inhibit dA phosphorylation by dGK. Taken together, these data suggest that under many conditions, dGK may be predominantly responsible for dG phosphorylation and that the cytosolic dCK may be more responsible for dA phosphorylation.

To measure phosphorylation of dA and dG under conditions in which both enzymes are present, we used an isolated perfused rat heart mode (Fig. 9, *A* and *B*). These data demonstrated that dA and dG were quite unstable when exposed to a whole tissue like the heart. Perfusion with [^3^H]-dG alone led to its rapid clearance from the perfusates into breakdown products (Fig. 9*A*). Addition of 2 μM I-H, a purine nucleotide phosphorylase inhibitor, largely prevented the breakdown of dG, but was not effective in preventing the breakdown of dA, which also required the addition of 5 μM EHNA to prevent deamination. Others have reported that deoxyadenosine is preferentially deaminated over phosphorylation in many tissues (43). Even in the presence of bothI-H and EHNA, about half of the parent dA was lost by 3 min and completely lost by 60 min. Conversely, ETV was completely stable during 60 min of heart perfusion, demonstrating that PNP is quite active in the heart and that ETV is not a substrate for this enzyme. Surprisingly, even though the parent compounds were unstable, phosphorylation of [^3^H]-dG was observed, even when added alone, but increased 3-fold when I-H was added. As expected, from our mitochondrial experiments, addition of 100 μM ETV inhibited [^3^H]-dG phosphorylation by 82.0 ± 0.7% (Fig. 9*B*, *p* < 0.0004). A small amount of phosphorylated [^3^H]-dA was also observed in the heart, but this amount was not inhibited by ETV (Fig. 9*B*). While the effects of ETV on dCK are unknown, it seems likely that [^3^H]-dA phosphorylation was mediated by dCK rather than dGK. Future work focused specifically on the cytoplasmic dCK will be required to clearly demonstrate the importance of dCK in dA and ETV phosphorylation, as well as the extent dCK maybe inhibited by ETV.

As we have reported earlier (23), exogenous TMP is not a substrate for phosphorylation in isolated mitochondria until it is broken down to thymidine. However, in the purine salvage pathway, both exogenous dAMP and dGMP are quite readily phosphorylated to dATP and dGTP (Figs. 7 and 8). dAMP is phosphorylated very quickly by the equilibrative enzymes adenylate kinase and diphosphokinase, indicating that both adenosine and deoxyadenosine are substrates for these enzymes. dGMP was also phosphorylated much more quickly than dG (Fig. 7), but its phosphorylation was not inhibited by Ap5A, indicating that this reaction is not catalyzed by adenylate kinase and is presumably phosphorylated by a mitochondrial monophosphate kinase with deoxyguanylate activity. The identity of this deoxymonophosphate kinase is not known but does not appear to be a function of the mitochondrial TMPK also known as CMPK/UMPK2 (44). These findings suggest that dAMP and dGMP arising from DNA turnover within the cell could be rapidly rephosphorylated and reused. However, these monophosphorylated forms are not likely to be transported across the plasma membrane unless first dephosphorylated to dA and dG, and therefore, these monophosphates are unlikely to serve as substrates when provided exogenously from outside the cell. However, human cells derived from individuals with TK2 and dGK deficiencies and cells from mouse models deficient in TK2 and dGK, as well as the mouse models themselves, have been treated with TMP and dGMP with varying degrees of improvement (7, 8, 20, 22, 45) in mtDNA depletion. Later work has demonstrated that the improvement is related to significant increases in dA, dG, dC, and thymidine concentrations with increased intracellular phosphorylation. dCK is likely responsible for dG phosphorylation in dGK deficiency (5), while increased residual activity of TK2 or low expression of TK1 may account for thymidine phosphorylation.

### Conclusions

The data in this paper demonstrate that intact functioning mitochondria from a variety of tissues phosphorylate dA, dG, and ETV. ETV competitively inhibits phosphorylation of dG, but not at levels that would be observed on treatment. However, ETV potently inhibits dA phosphorylation, which could affect the synthesis of dATP at ETV treatment levels and thus may be related to ETV toxicity. Further, dG itself is a potent inhibitor of dA phosphorylation, suggesting that dGK may play a more important role in dG phosphorylation than dA. Future work determining the role of the cytosolic dCK in phosphorylation of dG and dA should be undertaken.

## Experimental procedures

### Chemicals and biochemicals

Radioactive compounds including [^3^H]-dG (3000 DPM/pmol), [^3^H]-dA (3000 DPM/pmol), and [^3^H]-ETV (3000 DPM/pmol) were purchased from Moravek Biochemicals. ETV, adefovir, tenofovir, TDF, lobucavir, penciclovir, ganciclovir, and dideoxyguanosine were purchased from Toronto Research Chemicals. I-H (forodesine hydrochloride) was purchased from MedChemExpress. All other chemicals including EHNA and Ap5A were purchased from Sigma-Aldrich.

### Isolation of rat tissue mitochondria

Sprague-Dawley rats were raised in-house and used in accordance with the protocol approved by the Institutional Animal Care and Usage Committee. Tissue-specific (heart, liver, kidney, brain) mitochondria were isolated from adult female rats by using differential centrifugation as previously described (24, 46, 47). The viability and intactness of the mitochondria was confirmed by measuring the respiratory control ratio in a high-resolution respirometer (Oxygraph-2k) as previously described (24). Only mitochondria with a respiratory control ratio value of 6 or higher were used for experiments. The concentration of mitochondria was measured by the method of Lowry using bovine serum albumin as a standard. In earlier work, we demonstrated that this method of mitochondrial isolation yielded a product that was well washed and free of cytosolic contamination (40); thus, dCK is unlikely to be a contaminant.

### Mitochondrial incubation

Isolated tissue mitochondria were incubated at a concentration of 4 mg/ml when using [^3^H]-dG or [^3^H]-ETV as specified in figures in an incubation medium previously described (48). As the phosphorylation rate was much slower for [^3^H]-dA than for [^3^H]-dG or [^3^H]-ETV in all the tissues, we increased the mitochondria concentration to 8 mg/ml to observe phosphorylation of [^3^H]-dA. In our initial studies, dA and dG, while stable in heart mitochondria, were significantly degraded in mitochondria from the liver or kidney (Figs. S1 and S2). dG was subject to cleavage by PNP, which was effectively inhibited by I-H (2.0 μM [5 μM] (49)) (Fig. S1). dA was subject to deamination, which could be blocked by EHNA (5 μM) (50), an inhibitor of deamination (Fig. S2). To measure and compare phosphorylation and kinetics, a stable substrate concentration was necessary and EHNA (5 μM) (50) and I-H (2.0 μM) (49) were added to all mitochondrial incubations. These additions had no effect on phosphorylation in heart mitochondria but were effective in preventing the breakdown of dA and dG in mitochondria from the other tissues, allowing a comparison of tissue phosphorylation. The mitochondria were incubated *in vitro* for up to 2 h at 37 °C in a shaker bath. At the end of the incubation, reactions were terminated by addition of 10% TCA to an equal volume of incubation mixture (175 μl) and spun in a microcentrifuge to remove precipitates. The supernatant was neutralized with AG 11 A8 resin and filtered through a 0.2-μM nylon syringe filter. The neutralized and filtered supernatants were analyzed by ultra high pressure liquid chromatography (UPLC). Radioactivity was determined from aliquots of each sample and processed through a Beckman Coulter multipurpose liquid scintillation counter (model 6500).

### UPLC analysis

Extent of phosphorylation was measured and analyzed by UPLC (Agilent 1290) with a reverse-phase C18 column (Zorbax Eclipse Plus C18; 3.0 × 150 mm, 1.8 micron) coupled with an inline diode array to detect UV signal at 254 nm and to an inline liquid scintillation counter (β-Ram) to quantitate radioactivity. Standards were used to identify the parent and phosphorylated peaks of [^3^H]-dG, [^3^H]-ETV, and [^3^H]-dA.

### Langendorff heart perfusion

Rat hearts were perfused by Langendorff retrograde perfusion apparatus with [^3^H]-dG, [^3^H]-dA, or [^3^H]-ETV for 60 min under the conditions previously described (25, 26, 51). Perfused hearts were snap frozen into wafers by clamping the heart in liquid nitrogen, and the frozen tissue wafers were stored in −80 °C until processed as previously described for (24) UPLC analysis.

### Data analysis

Phosphorylated products of [^3^H]-dG, [^3^H]-ETV, and [^3^H]-dA in all isolated mitochondrial incubations included the monophosphate (∼20%), diphosphate (∼< 5%), and triphosphate (∼75%). The radioactivity in each of these peaks was quantitated and totaled to provide the total amount of radioactivity in the phosphorylated products for each labeled precursor. This total was divided by the specific radioactivity of the precursor and expressed as pmol/mg protein/hr. The K_ms_ were calculated from Lineweaver–Burk plots

$$\frac{1}{Vo}=\frac{K_{m}}{V_{max}\;\left[S\right]}+\;\frac{1}{V_{max}}$$

and Michaelis–Menten equation

$$\left(Vo=\frac{V_{max}\left[S\right]}{K_{m}+\left[S\right]}\right)$$

using the enzyme kinetics program in SigmaPlot, version 14.0. Where appropriate, the kinetic wizard in SigmaPlot (version 14.0) was used to determine the best fit of data to specific models of inhibition (competitive *versus* noncompetitive).

All numeric results in the text and figure legends represent the mean and standard error of the mean for at least 3 independent experiments. Deviations from this are described in the figure legends.

## Data availability

All data are contained within the manuscript.

## Supporting information

This article contains supporting information (49, 50).

## Conflict of interest

The authors declare that they have no conflicts of interest with the contents of this article.
