# Supplementary material for: Entecavir competitively inhibits deoxyguanosine and deoxyadenosine phosphorylation in isolated mitochondria and the perfused rat heart
Source: J Biol Chem. 2022 Mar 28;298(5):101876. doi: 10.1016/j.jbc.2022.101876 (PMC9097457; doi:10.1016/j.jbc.2022.101876)
Supplement: Supplemental Figures S1–S2 [file mmc1.docx]

**Entecavir competitively inhibits deoxyguanosine and deoxyadenosine phosphorylation in isolated mitochondria and the perfused rat heart.**

Avery S Ward^1^, Chia-Heng Hsiung^1, 2^, Daniel G Kesterson^1,3^, Vasudeva G Kamath^1, 4^ and Edward E McKee^1^

**Supplementary Material**:


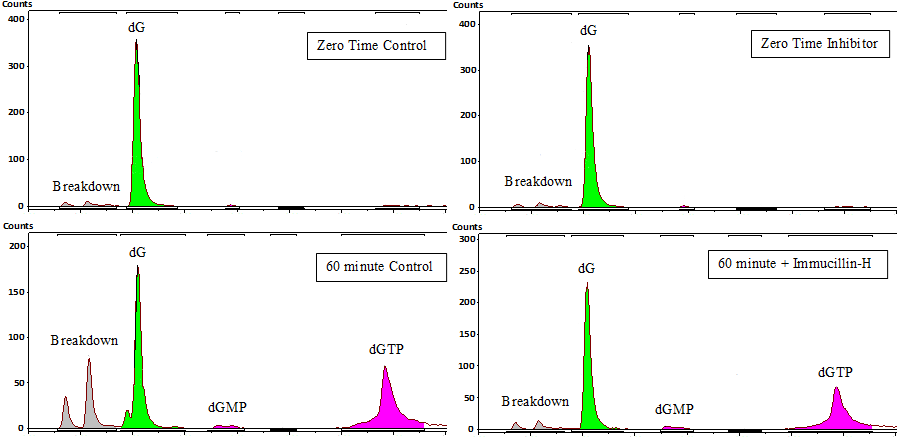
In our initial studies, dA and dG, while stable in heart mitochondria, were significantly degraded by deamination or cleavage by purine nucleoside phosphorylase (PNP) in mitochondria from liver or kidney. In order to measure phosphorylation and kinetics, and compare tissues, a stable substrate concentration of dA and dG was necessary. As shown in Figure 1, incubation of [^3^H]-dG for 60 min in mitochondria isolated from kidney resulted in the conversion of 22% of the [^3^H]-dG to breakdown products. The addition of Immucillin-H (2.0 μM) [40] (an inhibitor of purine nucleoside phosphorylase [1]) completely prevented this conversion (Figure 1 60min control versus 60 min + Immucillin-H).

**Figure 1:** Effect of immucillin-H to prevent the conversion of [^3^H]-dG to breakdown products. Kidney mitochondria were incubated as described in methods for 60 min with [^3^H]-dG and the presence and absence of Immucillin-H. Shown are UPLC chromatograms of deoxynucleoside/deoxynucleotide separation quantitated by an in-line scintillation counter (β-RAM II)

As shown in Figure 2, incubation of [^3^H]-dA for 60 min in mitochondria isolated from kidney resulted in the conversion of 28% of the [^3^H]-dA to breakdown products (Figure 2, 60 min control). The addition of Immucillin-H (2.0 μM) [40] (an inhibitor of purine nucleoside phosphorylase ]1]) had no effect on preventing [^3^H]-dA breakdown. However, 5 μM EHNA (a deaminase inhibitor [2]) completely prevented this conversion (Figure 2, 60min control versus 60 min + EHNA ). Immucillin-H and EHNA had no effect on [^3^H]-dA or [^3^H]-dG phosphorylation in heart mitochondria and was subsequently added to all
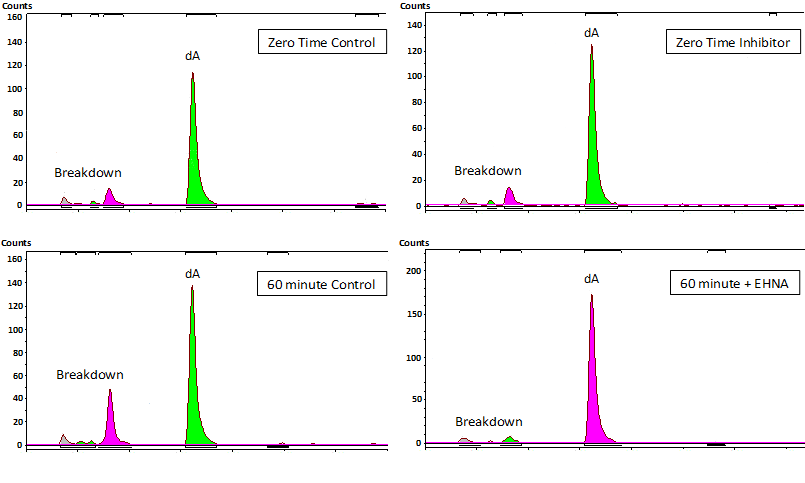
mitochondrial incubations.

**Figure 2:** Effect of EHNA to prevent the deamination of [^3^H]-dA to breakdown products. Liver mitochondria were incubated as described in methods for 60 min with [^3^H]-dA and the presence and absence of 5 μM EHNA. Shown are UPLC chromatograms of deoxynucleoside/deoxynucleotide separation quantitated by an in-line scintillation counter (β-RAM II)

**References:**

1. Kicska, G. A., Long, L., Hörig, H., Fairchild, C., Tyler, P. C., Furneaux, R. H., Schramm, V. L. and Kaufman, H. L. (2001) Immucillin H, a powerful transition-state analog inhibitor of purine nucleoside phosphorylase, selectively inhibits human T lymphocytes. Proc Natl Acad Sci U S A. **98**, 4593-4598

2. Podzuweit, T., Nennstiel, P. and Müller, A. (1995) Isozyme selective inhibition of cGMP-stimulated cyclic nucleotide phosphodiesterases by erythro-9-(2-hydroxy-3-nonyl) adenine. Cell Signal. **7**, 733-738
